# Bereitschaft zur COVID-19-Impfung unter Beschäftigten im Gesundheitswesen in Deutschland: Befragungsergebnisse aus dem Netzwerk Universitätsmedizin (NUM), November 2020 bis Januar 2021

**DOI:** 10.1007/s00103-021-03418-6

**Published:** 2021-09-23

**Authors:** Caterina Schug, Yesim Erim, Franziska Geiser, Nina Hiebel, Petra Beschoner, Lucia Jerg-Bretzke, Christian Albus, Kerstin Weidner, Susann Steudte-Schmiedgen, Andrea Borho, Marietta Lieb, Eva Morawa

**Affiliations:** 1 Psychosomatische und Psychotherapeutische Abteilung, Universitätsklinikum Erlangen, Friedrich-Alexander-Universität Erlangen-Nürnberg (FAU), Schwabachanlage 6, 91054 Erlangen, Deutschland; 2 Klinik und Poliklinik für Psychosomatische Medizin und Psychotherapie, Universitätsklinik Bonn, Bonn, Deutschland; 3 Klinik für Psychosomatische Medizin und Psychotherapie, Universitätsklinik Ulm, Ulm, Deutschland; 4 Klinik und Poliklinik für Psychosomatik und Psychotherapie, Universitätsklinik, Medizinische Fakultät, Universität zu Köln, Köln, Deutschland; 5 Klinik und Poliklinik für Psychotherapie und Psychosomatik, Universitätsklinikum C. G. Carus, Medizinische Fakultät, Technische Universität Dresden, Dresden, Deutschland

**Keywords:** COVID-19-Impfung, Gesundheitspersonal, Akzeptanz, Prädikatoren, Psychische Belastung, COVID-19-vaccination, Healthcare workers, Acceptance, Predictors, Mental burden

## Abstract

**Hintergrund:**

Die COVID-19-Pandemie stellt eine anhaltende Belastung für die Gesellschaft und das Gesundheitssystem dar. Die Bereitschaft des Gesundheitspersonals zur COVID-19-Impfung ist aufgrund seiner Schlüsselrolle in der Pandemiebewältigung besonders relevant.

**Ziel der Arbeit:**

Die Studie untersuchte die Impfbereitschaft des Gesundheitspersonals in Deutschland in Abhängigkeit von soziodemografischen, berufsbezogenen und COVID-19-spezifischen Merkmalen sowie psychischer Gesundheit.

**Methoden:**

Zwischen November 2020 und Januar 2021 wurden 6217 Beschäftigte im deutschen Gesundheitswesen mithilfe der Onlinebefragung VOICE im Rahmen des Netzwerks Universitätsmedizin (NUM) zu ihrer Impfbereitschaft, ihren soziodemografischen, berufsbezogenen, COVID-19-spezifischen und psychosozialen Daten befragt.

**Ergebnisse:**

Die Impfbereitschaft der Stichprobe lag bei 65,3 %. Mit einer höheren Impfbereitschaft assoziiert waren: männliches Geschlecht, Alter > 40 Jahre, keine Kinder und keinen Migrationshintergrund zu haben, keine Tätigkeit in der direkten Patientenversorgung, Zugehörigkeit zu einer COVID-19-Risikogruppe, Zugehörigkeit zur Berufsgruppe der Ärztinnen und Ärzte und der Psychologinnen und Psychologen im Vergleich mit den Pflegekräften, ausreichende Informiertheit über COVID-19 und der wahrgenommene Schutz durch die Maßnahmen nationaler/lokaler Behörden und des Arbeitgebers, Angst vor Infektion sowie keine Anzeichen von Depression. Die höchste Impfbereitschaft zeigten Ärztinnen und Ärzte.

**Diskussion:**

Die Bereitschaft zur COVID-19-Impfung im Gesundheitswesen war im beschriebenen Zeitraum insgesamt als mäßig einzuschätzen. Informationen über die Krankheit und Impfung, vor allem für jüngere, weibliche und nichtärztliche Beschäftigte, angemessene Schutzmaßnahmen und die Prävention depressiver Symptome könnten die Impfbereitschaft erhöhen.

## Abstract (en)

**Background:**

The COVID-19 pandemic is a continuing burden on society and the health system. The vaccination willingness among healthcare workers is of particular interest, as these groups play a key role in the pandemic response.

**Objectives:**

The present study investigated how the willingness of healthcare workers in Germany to get vaccinated depends on sociodemographic, occupational, and COVID-19-specific characteristics, as well as mental health.

**Methods:**

Between November 2020 and January 2021, 6217 employees in the German healthcare system were questioned about their vaccination willingness, sociodemographic, occupational, COVID-19-specific, and psychosocial characteristics using the online VOICE survey within the framework of the University Medicine Network (NUM).

**Results:**

The vaccination willingness of the sample group was 65.3%. A higher vaccination willingness was associated with male gender; age > 40 years; having no children or no migration background; not working in direct patient care; belonging to a COVID-19 risk group or professional group of physicians and psychologists in comparison with nursing staff; feeling sufficiently informed about COVID-19 and protected by the measures of the national or local authorities and the employer; fear of infection; and lack of signs of depression. Physicians showed the highest willingness to get vaccinated.

**Conclusions:**

During the study period, an overall moderate vaccination willingness against COVID-19 in the health sector was described. Information about the disease and vaccination, especially for younger people, females, and non-physicians, adequate protective measures and prevention of depressive symptoms could increase the vaccination willingness.

## Hintergrund

### Impfung gegen COVID-19

Bis Mitte Januar 2021 belief sich die Zahl der an Coronavirus Disease 19 (COVID-19) Erkrankten in Deutschland auf 2 Mio. mit ca. 47.600 an oder mit COVID-19 Verstorbenen. Eine Impfung gegen COVID-19 soll zum gesundheitlichen Schutz einzelner Personen und der Gesellschaft beitragen [1].

Am 08.01.2021 veröffentlichte die Ständige Impfkommission (STIKO) die erste COVID-19-Impf- sowie Priorisierungsempfehlung [2]. Zwischen dem 27.12.2020 und dem 16.06.2021 wurden in Deutschland 41.225.811 Erst- und 23.916.490 Zweitimpfungen (Impfquoten 49,6 % bzw. 28,8 %) mit Impfstoffen der Hersteller BioNTech/Pfizer, Moderna und AstraZeneca verabreicht [3]. In Deutschland [2] sowie beispielsweise in den USA [4] gehörten Beschäftigte im Gesundheitswesen zu den priorisierten Gruppen bei der Impfreihenfolge.

Durch die Förderung des Bundesministeriums für Bildung und Forschung (BMBF) wurde im April 2020 das nationale Netzwerk Universitätsmedizin (NUM) gegründet, um Maßnahmenpläne, Diagnostik- und Behandlungsstrategien der deutschen Universitätskliniken zu verbinden. Eines der NUM-Projekte ist egePan Unimed (Entwicklung, Testung und Implementierung von regional adaptiven Versorgungsstrukturen und Prozessen für ein evidenzgeleitetes Pandemiemanagement koordiniert durch die Universitätsmedizin). Ziel dieses Projektes ist es, Pandemiemanagementkonzepte in Deutschland und international abzustimmen, wissenschaftlich zu bewerten und zugunsten eines adäquaten regionalen Ressourcenmanagements zu integrieren. Da Impfungen nicht nur zur Eindämmung der Pandemie, sondern auch zur Beendigung der damit verbundenen Belastungen des Gesundheitssystems entscheidend beitragen [1], ist die Impfbereitschaft eng verbunden mit der körperlichen und mentalen Gesundheit der Beschäftigten in diesem Bereich [5, 6]. Impfbereitschaft und mentale Gesundheit wurden deshalb in der vorliegenden VOICE-Studie (Online-Survey zu Belastung und psychosozialen Ressourcen bei medizinischem Personal während der COVID-19-Pandemie) untersucht.

### Bereitschaft zur COVID-19-Impfung in der Allgemeinbevölkerung

In Deutschland verändert sich laut Befragungen von Universitäten und anderen Institutionen die Bereitschaft der Allgemeinbevölkerung zur COVID-19-Impfung dynamisch: Von April bis Ende des Jahres 2020 sank die Impfbereitschaft kontinuierlich von ca. 70–79 % [7, 8] auf 48 % [7]; seitdem stieg sie wieder auf 68 % Anfang März 2021 [7, 9, 10]. Mitte Juni 2021 gaben 31,4 % der Ungeimpften in Deutschland an, sich auf jeden Fall impfen lassen zu wollen, 29,7 % hingegen wollten dies auf keinen Fall. Geimpfte und Impfwillige bis 74 Jahre ergäben zusammengerechnet eine Impfquote von ca. 76 % [7].

Im April 2020 betrug die Impfbereitschaft in der Allgemeinbevölkerung von 7 europäischen Ländern insgesamt 73,9 % [8], wobei Frankreich die niedrigste (62 %), Dänemark die höchste (80 %) und Deutschland eine mittlere (70 %) Impfbereitschaft aufwies. Von 19 im Juni 2020 untersuchten Ländern wurde in China die höchste (fast 90 %) und in Russland die niedrigste (unter 55 %) Impfbereitschaft konstatiert [11]. In den USA nahm die Impfbereitschaft von April bis Oktober 2020 von 71 % auf 53,6 % ab [12].

Am 21.06.2021 verzeichnete Frankreich eine Erstimpfungsquote von ca. 50 %, die USA von 47 % und China von ca. 71 % [13].

### Welche Faktoren beeinflussen die Bereitschaft zur COVID-19-Impfung?

Laut einer deutschen Erhebung ist das am häufigsten genannte Motiv für eine Impfung die Bewahrung der eigenen Gesundheit (52 % der Befragten). 24 % sehen die Impfung vor allem als ein Mittel, um pandemiebedingte Einschränkungen wie die Kontaktreduktion zu vermeiden [7]. Gründe für die Zögerlichkeit in Bezug auf eine COVID-19-Impfung sind die Sorge vor Nebenwirkungen (55 %) und davor, die COVID-19-Impfung könnte nicht sicher sein (15 %; [8]).

Demografische Faktoren, die in bisherigen Studien mit einer höheren Impfbereitschaft einhergingen, waren männliches Geschlecht [7, 8, 12, 14, 15], höheres Alter [7, 10, 12, 16, 17], höhere formale Bildung [16, 18], höheres Einkommen [12], Verheiratetenstatus [14] und Kinderlosigkeit [15].

Einen wichtigen Faktor für die Impfbereitschaft stellt außerdem das Vertrauen in die Sicherheit der Impfung dar [7, 15, 16]. Anfang Februar 2021 hielten 57 % der in Deutschland Befragten eine COVID-19-Impfung für sicher [9], nachdem von Mai bis Dezember 2020 die Zustimmung dazu, dass ein COVID-19-Impfstoff sicher ist, auf einer Skala von 1 = „stimme überhaupt nicht zu“ bis 7 = „stimme voll und ganz zu“ von 4,4 auf 3,7 abgesunken war [7].

Sowohl Personen mit einem höheren Vertrauen in die Wissenschaft [10, 19] als auch solche, die sich mehr informieren [9] und sich besser informiert fühlen [7, 17], sind eher zu einer COVID-19-Impfung bereit. Mitte Juni 2021 gaben 55 % der Ungeimpften in Deutschland an, sich sehr häufig zum Thema COVID-19 zu informieren [7]. Fehlende Information wird als Grund angegeben, sich nicht impfen zu lassen [16].

Außerdem besteht ein positiver Zusammenhang zwischen der Zufriedenheit der Befragten mit politischen Entscheidungen hinsichtlich der COVID-19-Pandemie [10] sowie der wahrgenommenen Angemessenheit der Infektionsschutzmaßnahmen [7] und der Bereitschaft zur COVID-19-Impfung. Sowohl die Motivation, sich zum Thema COVID-19 zu informieren, als auch das Vertrauen in staatliche Maßnahmen stiegen in der Allgemeinbevölkerung von März bis Juli 2020 zunächst, sanken dann jedoch im Verlauf dieses Zeitraumes wieder [20]. Auch das Vertrauen in Institutionen wie das Robert Koch-Institut, das unter anderem mit höherer Akzeptanz von Infektionsschutzmaßnahmen einherging, sank im Verlauf der ersten Pandemiemonate leicht [21].

Weitere Faktoren, die die Bereitschaft zur COVID-19-Impfung begünstigen, sind: Akzeptanz der Grippeimpfung (z. B. [15]), sich nicht auf die COVID-19-Impfbereitschaft anderer verlassen zu wollen, die Wahrnehmung von Impfen als Bürgerpflicht, Impfbefürwortung in der eigenen Familie, die Angst vor einer Severe-Acute-Respiratory-Syndrome-Corona-Virus-2-(SARS-CoV-2-)Infektion, das Anstreben einer Impfung aus Gesundheitsgründen [7] sowie die wahrgenommene Gefährlichkeit von COVID-19 für andere [17] und sich selbst [15].

Dem entgegen stehen individuelle Nutzen- und Risiko-Abwägungen bezüglich der Impfung oder die Annahme, dass Impfnebenwirkungen verheimlicht werden [7]. Beide Faktoren führen zu einer geringeren Impfbereitschaft.

### Bereitschaft zur COVID-19-Impfung unter Beschäftigten im Gesundheitswesen

Die Bereitschaft zur COVID-19-Impfung unter Beschäftigten im Gesundheitswesen ist von besonderer Relevanz: Einerseits ist diese Berufsgruppe durch Patientenkontakte deutlich mehr exponiert, andererseits ist ihre Gesundheit für die Stabilität des Gesundheitssystems während der Pandemie von hoher Bedeutung. Beschäftigte im Gesundheitswesen zeigten im Vergleich mit der Allgemeinbevölkerung in Deutschland tendenziell eine etwas geringere COVID-19-Impfbereitschaft ([7], Welle 29). In Südostasien [22] und China [23] war die Impfbereitschaft unter Beschäftigten im Gesundheitswesen höher als in der Allgemeinbevölkerung, in Israel wurde bei einer Befragung im März 2020 diesbezüglich kein Unterschied gefunden [15].

In den Vereinigten Staaten waren laut Unroe et al. [24] im November 2020 45 % des medizinischen Personals dazu bereit, sich sobald wie möglich gegen COVID-19 impfen zu lassen. Die Befürchtung von Nebenwirkungen war der Hauptgrund für eine Ablehnung der Impfung (70 %), gefolgt von gesundheitlichen Sorgen (34 %), Zweifel an der Effektivität der Impfung (20 %) und religiösen Gründen (12 %). Unter medizinischem Personal in Frankreich lag die COVID-19-Impfbereitschaft zwischen März und Juli 2020 bei 76,9 % [25], in Malta im September 2020 bei 55 % [26] und in der Türkei bei 68,6 % [27].

Auch unter Beschäftigten im Gesundheitswesen korrelieren männliches Geschlecht [24–27] und höheres Alter [25] bzw. ein Alter über 60 Jahre [24] mit höherer Bereitschaft zur COVID-19-Impfung. Außerdem stehen COVID-19-bedingte Angst [25], die wahrgenommene eigene Ansteckungsgefahr [23], stärkeres kollektives Verantwortungsgefühl, stärkeres Vertrauen in die Impfung [28] sowie eine höhere generelle Impfbereitschaft [25], eine (angestrebte) Grippeimpfung im vorigen [25] oder nächsten Winter [26] und direkter Kontakt zu SARS-CoV-2-Infizierten [15] in Zusammenhang mit einer höheren COVID-19-Impfbereitschaft. Unter Ärztinnen und Ärzten ist die COVID-19-Impfbereitschaft am höchsten verglichen mit anderen medizinischen Berufsgruppen [25, 26].

Nur ca. die Hälfte des Anfang Dezember 2020 befragten medizinischen Personals in Deutschland hielt sich für ausreichend über die COVID-19-Impfung informiert, um über sie Auskunft geben zu können. Beschäftigte im Gesundheitswesen bevorzugten häufiger als alle anderen Berufsgruppen den Arbeitsplatz und das Gesundheitsamt als potenziellen Impfort [7].

Die vorliegende Studie zur Impfbereitschaft unter Beschäftigten im Gesundheitswesen in Deutschland soll folgende Fragen beantworten:

1. Wie hoch ist die Bereitschaft zur COVID-19-Impfung bei Beschäftigten im Gesundheitswesen in Deutschland?
2. Wie ist die Bereitschaft zur COVID-19-Impfung in Abhängigkeit von soziodemografischen, berufsbezogenen und COVID-19-spezifischen Charakteristika ausgeprägt?
3. Wie hängt die Bereitschaft zur COVID-19-Impfung mit psychischer Gesundheit und psychischen Ressourcen zusammen?
4. Welche Charakteristika sind statistisch signifikante Prädiktoren für die COVID-19-Impfbereitschaft unter Beschäftigten im Gesundheitswesen in Deutschland?

## Methoden

Zwischen dem 18.11.2020 und dem 07.01.2021 fand der zweite Messzeitpunkt der webbasierten prospektiven VOICE-Studie statt, welcher den vorliegenden Analysen zugrunde liegt. Die psychosomatischen Abteilungen der Universitätskliniken Erlangen, Bonn, Ulm, Köln und Dresden teilten den Link über Onlineplattformen oder Mailinglisten für die Mitarbeitenden der Universitätskliniken und mehrerer kommunaler Krankenhäuser sowie über Fachverbände und sonstige Netzwerke.

Die 15-minütige Umfrage wurde mit den Softwaretools für wissenschaftliche Onlinebefragungen Unipark und SoSci programmiert. Einschlusskriterien waren ein Mindestalter von 18 Jahren, Berufstätigkeit im Gesundheitswesen, Arbeitsort in Deutschland und ausreichende Deutschkenntnisse. Insgesamt 6217 Befragte beantworteten den Fragebogen vollständig und wurden in die vorliegende Studie eingeschlossen. Mehrfache Teilnahmen derselben Person (identifizierbar durch einen von den Befragten erstellten Code) wurden gelöscht.

### Fragebögen

Die Teilnehmenden wählten zu der Aussage: „Sobald es einen in Deutschland zugelassenen Impfstoff gegen COVID-19 gibt, werde ich mich impfen lassen“, eine der vorgegebenen Antwortmöglichkeiten „Ja“ oder „Nein“.

Folgende soziodemografischen Daten wurden erhoben: Geschlecht, Alterskategorie (18–30, 31–40, 41–50, 51–60, 61–70, > 70 Jahre), alleinlebend (Ja/Nein), Kinder (Ja/Nein) und Migrationshintergrund (Ja/Nein; lag vor, wenn der/die Teilnehmende oder mindestens ein Elternteil die deutsche Staatsangehörigkeit nicht durch Geburt erworben hat). Erhobene berufliche Merkmale waren Arbeitsort, Beruf, Berufserfahrung und Beschäftigung in Voll- oder Teilzeit.

Depressionssymptome wurden anhand des Ultrakurz-Screening-Instruments für Depression des Patient Health Questionnaire (PHQ‑2; [29]) auf einer vierstufigen Likert-Skala von 0 = „überhaupt nicht“ bis 3 = „fast jeden Tag“ gemessen. Summenscores ≥ 3 werden als wahrscheinliche klinisch relevante Fälle von Depression angesehen [30]. Cronbachs Alpha für den PHQ‑2 betrug 0,77.

Angstsymptome wurden mithilfe des Ultrakurz-Screening-Instruments für generalisierte Angststörungen (engl. Generalized Anxiety Disorder; GAD‑2; [29]) auf einer vierstufigen Likert-Skala von 0 = „überhaupt nicht“ bis 3 = „fast jeden Tag“ gemessen. Summenscores ≥ 3 werden als wahrscheinliche klinisch relevante Fälle von generalisierter Angst angesehen [31]. Cronbachs Alpha für GAD‑2 war 0,79.

Das Kohärenzgefühl wurde mit 3 Items der deutschen Version der Sense of Coherence Scale (SOC; [32]) auf einer siebenstufigen Likert-Skala von 1 = „sehr oft“ bis 7 = „sehr selten oder nie“ erhoben. Cronbachs Alpha war 0,70.

Die Angst vor einer SARS-CoV-2-Infektion wurde mit 2 selbst erstellten Items („Ich hatte Angst, mich anzustecken“ und „Ich hatte Angst, Verwandte oder meine Familie anzustecken“) auf einer fünfstufigen Likert-Skala von 0 = „stimme überhaupt nicht zu“ bis 4 = „stimme vollständig zu“ (bezogen auf die letzten 2 Wochen) gemessen.

Der Grad der Informiertheit über COVID-19 und das Gefühl des Vorbereitetseins auf die Pandemie wurden erhoben, indem bei den folgenden 2 Aussagen die Zustimmung fünfstufig von 0 = „stimme überhaupt nicht zu“ bis 4 = „stimme vollständig zu“ erfragt wurde: „Ich fühle mich über COVID-19 ausreichend informiert“ und „Ich fühle mich jetzt besser vorbereitet auf die Pandemie als im Frühjahr.“

Außerdem wurden mit je einem Item erfasst: Abteilungswechsel während der Pandemie (Ja/Nein), direkter Kontakt zu SARS-CoV-2-Infizierten (durch einen Test nachgewiesen) bei der Arbeit (Ja/Nein), Kontakt mit kontaminiertem Material während der Arbeit (Ja/Nein), Zugehörigkeit zu einer Risikogruppe aufgrund des Alters oder einer chronischen Erkrankung (Auswahl keiner, einer oder beider Optionen war möglich), eigene SARS-CoV-2-Infektion (Ja/Nein/Ich weiß nicht), Grad der Arbeitsbelastung der Abteilung (von 0 = „stark unterdurchschnittlich“ bis 4 = „stark überdurchschnittlich“) und Arbeit im Homeoffice (Ja, ausschließlich/Ja, teilweise/Nein).

### Statistische Auswertung

Mithilfe von SPSS V.24 wurden neben absoluten und relativen Häufigkeiten Chi-Quadrat-Tests mitsamt der Effektstärkemaße Cramers V bzw. Phi (≥ 0,1 = kleine, ≥ 0,3 = mittlere und ≥ 0,5 = große Effektstärke) sowie eine binäre logistische Regression berechnet. Signifikanzentscheidungen basierten auf dem Alphafehlerniveau von 0,05.

In die Regressionsanalyse wurden alle zuvor beschriebenen Variablen eingeschlossen, bis auf Berufserfahrung, Beschäftigungsmodell (Vollzeit/Teilzeit) und eigene SARS-CoV-2-Infektion. Faktoren, zu deren Erhebung Zustimmungsgrade erfragt wurden, wurden dichotomisiert („stimme vollständig zu“ und „stimme zu“ als eine Kategorie).

## Ergebnisse

Merkmale der Stichprobe von Beschäftigten im Gesundheitswesen in Deutschland finden sich in Tab. 1. Ein Großteil der Stichprobe (73,3 %) war weiblich, was annähernd repräsentativ für das Gesundheitspersonal in Deutschland ist (75,6 % [33]). Das Alter der Teilnehmenden verteilte sich relativ gleichmäßig auf die 4 Altersgruppen von 18 bis 60 Jahren (< 30 Jahre: 22,6 % der Stichprobe vs. 15,4 % des gesamten Gesundheitspersonals; 31–40 Jahre: 24,1 % vs. 21,6 %; 41–50 Jahre: 22,3 % vs. 22,4 %; 51–60 Jahre: 24,3 % vs. 28,3 %), 6,7 % waren über 60 Jahre alt (vs. 12,2 %). Der Anteil an Ärztinnen und Ärzten (24,8 % vs. 8 %) und Pflegekräften (23,2 % vs. 11,3 %) in der Stichprobe war höher als beim Gesundheitspersonal in Deutschland. Der Anteil der in der Arzt- und Praxishilfe Tätigen (z. B. medizinische Fachangestellte) und Laborassistentinnen und -assistenten war annähernd repräsentativ (13,4 % vs. 13,9 % im gesamten Gesundheitspersonal). 81,2 % der Stichprobe (vs. 64,2 %) hatten direkten Patientenkontakt und 36,5 % (vs. 42 %) arbeiteten in Teilzeit [33].

**Table 1:** 

	Gesamtstichprobe *N* = 6217	Impfbereitschaft *n* (%)	*p*-Wert (Effektstärke: Phi/Cramer V)
*Geschlecht, n (%)*	–	–	**<** **0,001 (0,145)**
Frauen	4556 (73,3)	2789 (61,2)	
Männer	1641 (26,4)	1259 (76,7)	**<** **0,001 (0,144)**^**b**^
Divers	20 (0,3)	9 (45,0)	
*Alter, Jahre, n (%)*	–	–	**<** **0,001 (0,080)**
18–30	1406 (22,6)	853 (60,7)	–
31–40	1496 (24,1)	948 (63,4)	–
41–50	1386 (22,3)	913 (65,9)	–
51–60	1513 (24,3)	1029 (68,0)	–
> 60	416 (6,7)	314 (75,5)	–
*Alleinlebend, n (%)*	–	–	**0,030 (0,027)**
Ja	1489 (24,0)	937 (62,9)	**–**
Nein	4728 (76,0)	3120 (66,0)	–
*Kinder, n (%)*	–	–	0,892 (0,002)
Ja	3156 (50,8)	2064 (65,3)	–
Nein	3058 (49,2)	1993 (65,2)	–
*Migrationshintergrund, n (%)*	–	–	**0,001 (0,043)**
Ja	701 (11,3)	417 (59,5)	–
Nein	5516 (88,7)	3640 (66,0)	–
*Versorgung/Pflege kranker Angehöriger, n (%)*	–	–	0,846 (0,002)
Ja (im eigenen Haushalt/nicht im eigenen Haushalt)	1014 (16,3)	659 (65,0)	–
Nein	5203 (83,7)	3398 (65,3)	–
*Beruf, n (%)*	–	–	**<** **0,001 (0,162)**
Arzt/Ärztin	1541 (24,8)	1181 (76,6)	–
Pflegekraft	1444 (23,2)	822 (56,9)	–
MTA (Medizinisch-technische Angestellte)^a^	831 (13,4)	487 (58,6)	–
Psychologe/in, psychologische/r Psychotherapeut/in	446 (7,2)	305 (68,4)	–
Nichtmedizinische Heilberufe (z. B. Physio‑/Ergo‑/Sprachtherapeut/in, Heil‑/Sozialpädagoge/in)	438 (7,0)	263 (60,0)	–
Verwaltungsmitarbeiter/in mit/ohne direkten Patientenkontakt	400 (6,5)	242 (60,5)	–
Student/in, Auszubildende/r	156 (2,5)	109 (69,9)	–
Forschung	98 (1,6)	69 (70,4)	–
Sonstiges (z. B. Seelsorger/in, Lehrkraft, Sozialarbeiter/in, IT-/Technikmitarbeiter/in)	863 (13,9)	579 (67,1)	–
*Berufserfahrung im Bereich der Patientenversorgung, n (%)*	–	–	**0,050 (0,035)**
< 3 Jahre	666 (10,7)	429 (64,4)	–
3–6 Jahre	797 (12,8)	494 (62,0)	–
> 6 Jahre	3583 (57,6)	2338 (65,3)	–
Nicht in der Patientenversorgung tätig	1171 (18,8)	796 (68,0)	–
*Erwerbstätigkeit, n (%)*	–	–	0,055 (0,024)
Vollzeit	3965 (63,8)	2622 (66,1)	–
Teilzeit	2252 (36,2)	1435 (63,7)	–
*Infektion mit SARS-CoV‑2, n (%)*	–	–	**<** **0,001 (0,105)**
Ja	147 (2,4)	92 (62,6)	–
Nein	4352 (70,0)	2981 (68,5)	–
Ich weiß nicht	1718 (27,6)	984 (57,3)	–
*Kontakt mit SARS-CoV-2-infizierten Patienten/Patientinnen bei der Arbeit, n (%)*	–	–	0,718 (0,005)
Ja	2699 (43,4)	1768 (65,5)	–
Nein	3518 (56,6)	2289 (65,1)	–
*Kontakt mit durch SARS-CoV‑2 kontaminiertem Material bei der Arbeit, n (%)*	–	–	0,711 (0,005)
Ja	2042 (32,8)	1326 (64,9)	–
Nein	4175 (67,2)	2731 (65,4)	–
*Risikogruppe, n (%)*	–	–	**<** **0,001 (0,051)**
Ja	1785 (28,7)	1233 (69,1)	–
Nein	4432 (71,3)	2824 (63,7)	–
*Auslastung der Abteilung, n (%)*	–	–	0,476 (0,024)
Stark unterdurchschnittlich	178 (2,9)	109 (61,2)	–
Leicht unterdurchschnittlich	679 (10,9)	446 (65,7)	–
Durchschnittlich	2086 (33,6)	1349 (64,7)	–
Leicht überdurchschnittlich	1763 (28,4)	1176 (66,7)	–
Stark überdurchschnittlich	1511 (24,3)	977 (64,7)	–
*Wechsel des Arbeitsbereiches seit Beginn der COVID-19-Pandemie, n (%)*	–	–	**0,036 (0,027)**
Ja	913 (14,7)	568 (62,2)	–
Nein	5304 (85,3)	3489 (65,8)	–
*Homeoffice, n (%)*	–	–	**0,001 (0,042)**
Ja (ausschließlich/teilweise)	940 (15,1)	658 (70,0)	–
Nein	5277 (84,9)	3399 (64,4)	–

Signifikante *p*-Werte und korrespondierende Effektstärken sind fett markiert

^a^Die Gruppe setzte sich aus folgenden beruflichen Untergruppen zusammen: Medizinische Fachangestellte, Medizinisch-technische Laboratoriumsassistentinnen und -assistenten, Medizinisch-technische Radiologieassistentinnen und -assistenten, Pharmazeutisch-technische Assistentinnen und Assistenten

^b^Diverse aus Analyse ausgeschlossen

2,4 % waren zum Befragungszeitpunkt selbst mit SARS-CoV‑2 infiziert gewesen. 43,4 % hatten direkten Kontakt zu COVID-19-Patientinnen und -Patienten.

### Impfbereitschaft unter den befragten Beschäftigten im Gesundheitswesen und im Vergleich mit der Allgemeinbevölkerung

Die Impfbereitschaft der Stichprobe lag mit 65,3 % höher als die der Allgemeinbevölkerung in diesem Zeitraum (Vergleichswert von 50 % von Anfang Dezember 2020 [7]).

Männer wiesen eine signifikant höhere Impfbereitschaft auf als Frauen (76,7 % vs. 61,2 %; *p* < 0,001, Phi = 0,144) und Ärztinnen und Ärzte (76,6 %) die höchste von allen befragten Berufsgruppen (*p* < 0,001, Cramer V = 0,162). Nichtinfizierte zeigten eine höhere Impfbereitschaft (68,5 %) als diejenigen, die bereits positiv auf SARS-CoV‑2 getestet waren (62,6 %) oder angaben, es nicht zu wissen (57,3 %; *p* < 0,001, Cramer V = 0,105). Personen, die sich aufgrund von Alter oder Vorerkrankung in einer Risikogruppe für einen schweren COVID-19-Verlauf sahen (*p* ≤ 0,001; Phi = 0,051), die seit Beginn der COVID-19-Pandemie den Arbeitsbereich nicht gewechselt hatten (*p* = 0,036; Phi = 0,027), und diejenigen, die teilweise oder ausschließlich im Homeoffice arbeiteten (*p* = 0,001; Phi = 0,042), zeigten eine signifikant höhere Impfbereitschaft.

Alleinlebende (*p* = 0,03; Phi = 0,027), Beschäftigte mit Migrationshintergrund (*p* = 0,001; Phi = 0,043) und Beschäftigte in der direkten Patientenversorgung (*p* = 0,03; Phi = 0,028) zeigten eine niedrigere Impfbereitschaft.

### Impfbereitschaft in Abhängigkeit von Informiertheit, wahrgenommenem Schutz durch Maßnahmen, Angst vor Ansteckung und mentaler Gesundheit

Knapp 70 % der Befragten stimmten eher oder vollständig der Aussage zu, über COVID-19 ausreichend informiert zu sein. Weniger als die Hälfte (47,6 %) fühlten sich zum Zeitpunkt der Befragung besser auf die Pandemie vorbereitet als im Frühjahr 2020. 27,1 % der befragten Beschäftigten stimmten eher oder vollständig zu, sich durch Maßnahmen nationaler und lokaler Behörden und 43,5 % durch Maßnahmen des Arbeitgebers geschützt zu fühlen. 40,2 % stimmten eher oder vollständig der Aussage zu, Angst vor einer eigenen Ansteckung zu haben, und 61,1 % hatten Angst davor, die Familie anzustecken.

**Figure 1:**
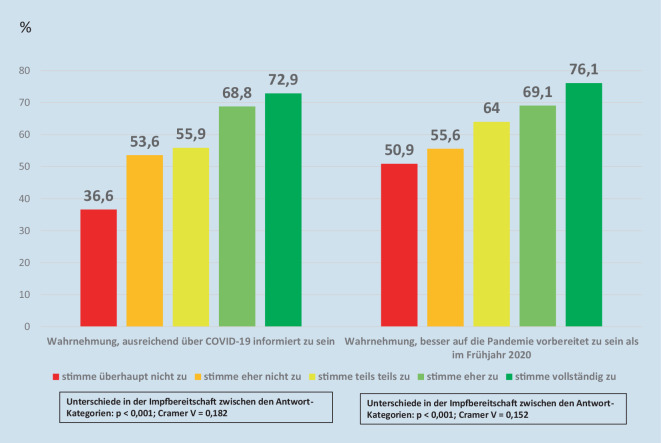

Stärkere Zustimmung dazu, sich ausreichend über COVID-19 informiert zu fühlen (*p* < 0,001; Cramer V = 0,182) und besser auf die Pandemie vorbereitet zu sein als im Frühjahr (*p* < 0,001; Cramer V = 0,152), war mit einer größeren Impfbereitschaft assoziiert (Abb. 1).

**Figure 2:**
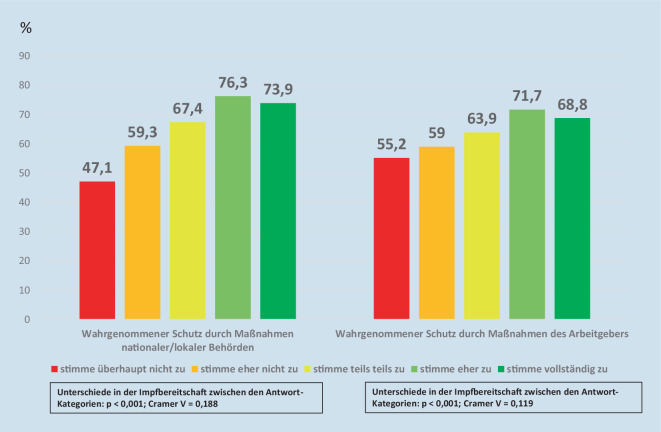

Eine höhere Impfbereitschaft ging auch einher mit einem besseren wahrgenommenen Schutz durch die Maßnahmen nationaler und lokaler Behörden (*p* < 0,001; Cramer V = 0,188) und durch Maßnahmen des Arbeitgebers (*p* < 0,001; Cramer V = 0,119; Abb. 2).

Größere Angst davor, sich selbst (*p* < 0,001; Cramer V = 0,161) und Angehörige zu infizieren (*p* < 0,001; Cramer V = 0,113), war ebenfalls mit einer größeren Impfbereitschaft assoziiert.

Kein Verdacht auf eine klinisch relevante Depression (PHQ-2 < 3; *p* < 0,001; Phi = 0,058) und kein Verdacht auf klinisch relevante generalisierte Angst (GAD-2 < 3; *p* = 0,005; Phi = 0,035) sowie ein stärkeres Kohärenzgefühl (SOC-3 > 15; *p* < 0,001; Phi = 0,058) hingen laut Chi-Quadrat-Tests mit einer signifikant höheren Impfbereitschaft zusammen*.*

### Ergebnisse der Regressionsanalyse

Die binäre logistische Regression mit Impfbereitschaft als Kriteriumsvariable klärte 13,5 % (Nagelkerkes R‑Quadrat) der Varianz auf. Signifikante Faktoren, die mit einer höheren Chance, sich impfen zu lassen, zusammenhingen, waren: männliches Geschlecht, Altersgruppen 41–50 und > 50 Jahre (verglichen mit 18–30 Jahren), keine Kinder und keinen Migrationshintergrund zu haben, keine Tätigkeit in der direkten Patientenversorgung, Zugehörigkeit zu einer COVID-19-Risikogruppe, zur Berufsgruppe der Ärztinnen und Ärzte und der Psychologinnen und Psychologen (im Vergleich mit dem Pflegepersonal), sich ausreichend über COVID-19 informiert und durch die Maßnahmen nationaler/lokaler Behörden und des Arbeitgebers geschützt zu fühlen, Angst, sich selbst oder Angehörige zu infizieren, und keine klinisch bedeutsamen Depressionssymptome (PHQ-2 < 3) zu zeigen (Tab. 2). Eine generalisierte Angst und Kohärenzerleben sowie direkter Kontakt zu COVID-19-Patienteninnen und -Patienten während der Arbeit wiesen hier keine signifikant erhöhte Chance für Impfbereitschaft auf.

**Table 2:** 

Unabhängige Variablen	Pseudo R^2^ = 13,5 % (Nagelkerke); Hosmer-Lemeshow = 7,198; df = 8; *p* = 0,515; 2‑Log-Likelihood = 7360,731							
	Regressionskoeffizient B	Standardfehler	Wald	df	*p*	OR	95 % CI: unterer Wert	95 % CI: oberer Wert
*Geschlecht*^b^* (Frauen* *=* *Ref.)*								
Männer	0,670	0,073	85,216	1	**<** **0,001**	**1,954**	**1,695**	**2,253**
*Alter (18–30 Jahre* *=* *Ref.)*								
31–40	0,035	0,087	0,161	1	0,688	1,036	0,873	1,228
41–50	0,255	0,097	6,858	1	**0,009**	**1,290**	**1,066**	**1,560**
> 50	0,309	0,102	9,237	1	**0,002**	**1,361**	**1,116**	**1,661**
*Alleinlebend (Ja* *=* *Ref.)*								
Nein	0,087	0,070	1,551	1	0,213	1,091	0,951	1,250
*Kinder (Ja* *=* *Ref.)*								
Nein	0,309	0,073	17,742	1	**<** **0,001**	**1,362**	**1,180**	**1,573**
*Migrationshintergrund (Ja* *=* *Ref.)*								
Nein	0,260	0,088	8,772	1	**0,003**	**1,297**	**1,092**	**1,541**
*Versorgung/Pflege kranker Angehöriger (Ja* *=* *Ref.)*								
Nein	0,015	0,079	0,035	1	0,852	1,015	0,869	1,185
*Beruf (Pflegekraft* *=* *Ref.)*								
Arzt/Ärztin	0,793	0,087	83,405	1	**<** **0,001**	**2,209**	**1,864**	**2,619**
MTA^a^	0,039	0,097	0,163	1	0,687	1,040	0,860	1,257
Psychologe/in, Psychotherapeut/in	0,476	0,126	14,248	1	**<** **0,001**	**1,610**	**1,257**	**2,062**
Nichtmedizinische Heilberufe	0,050	0,121	0,168	1	0,682	1,051	0,829	1,331
Verwaltungsmitarbeiter/in	−0,008	0,132	0,004	1	0,952	0,992	0,766	1,285
Sonstiges	0,228	0,100	5,266	1	**0,022**	**1,257**	**1,034**	**1,527**
*Patientenversorgung (Ja* *=* *Ref.)*								
Nein	0,260	0,088	8,677	1	**0,003**	**1,297**	**1,091**	**1,542**
*Erwerbstätigkeit (Vollzeit* *=* *Ref.)*								
Teilzeit	0,046	0,065	0,485	1	0,486	1,047	0,921	1,190
*Kontakt mit SARS-CoV-2-infizierten Patienten/Patientinnen bei der Arbeit (Ja* *=* *Ref.)*								
Nein	0,034	0,076	0,201	1	0,654	1,035	0,892	1,200
*Kontakt mit durch SARS-CoV‑2 kontaminiertem Material bei der Arbeit (Ja* *=* *Ref.)*								
Nein	−0,064	0,078	0,670	1	0,413	0,938	0,804	1,094
*Risikogruppe (Nein* *=* *Ref.)*								
Ja	0,149	0,071	4,432	1	**0,035**	**1,161**	**1,010**	**1,334**
*Wechsel des Arbeitsbereiches seit Beginn der COVID-19-Pandemie (Ja* *=* *Ref.)*								
Nein	0,148	0,080	3,436	1	0,064	1,160	0,992	1,357
*Homeoffice (Nein* *=* *Ref.)*								
Ja	0,049	0,085	0,326	1	0,568	1,050	0,888	1,241
*Ausreichende Informiertheit über COVID-19*^c^								
Stimme eher/vollständig zu	0,556	0,063	78,533	1	**<** **0,001**	**1,745**	**1,543**	**1,973**
*Bessere Vorbereitung auf die Pandemie als im Frühjahr*^c^								
Stimme eher/vollständig zu	0,118	0,063	3,514	1	0,061	1,125	0,995	1,273
*Sich geschützt fühlen durch Maßnahmen nationaler/lokaler Behörden*^c^								
Stimme eher/vollständig zu	0,496	0,072	47,928	1	**<** **0,001**	**1,642**	**1,427**	**1,889**
*Sich geschützt fühlen durch Maßnahmen des Arbeitgebers*^c^								
Stimme eher/vollständig zu	0,166	0,064	6,856	1	**0,009**	**1,181**	**1,043**	**1,337**
*Angst, sich zu infizieren*^c^								
Stimme eher/vollständig zu	0,368	0,068	29,519	1	**<** **0,001**	**1,445**	**1,266**	**1,651**
*Angst, Familie/Angehörige zu infizieren*^c^								
Stimme eher/vollständig zu	0,344	0,068	25,677	1	**<** **0,001**	**1,410**	**1,235**	**1,611**
*Depressivität (≥* *3* *=* *Ref.)*								
< 3	0,177	0,076	5,392	1	**0,020**	**1,194**	**1,028**	**1,386**
*Generalisierte Angst (≥* *3* *=* *Ref.)*								
< 3	−0,001	0,079	**<** 0,001	1	0,988	0,999	0,856	1,166
*Kohärenzgefühl (≤* *15* *=* *Ref.)*								
> 15	0,058	0,064	0,824	1	0,364	1,060	0,935	1,201

Signifikante *p*-Werte und korrespondierende ORs und CI sind fett markiert

*OR* Odds Ratio, *CI* Konfidenzintervall, *Ref.* Referenzgruppe

^a^Die Gruppe setzte sich aus folgenden beruflichen Untergruppen zusammen: Medizinische Fachangestellte, Medizinisch-technische Laboratoriumsassistentinnen und -assistenten, Medizinisch-technische Radiologieassistentinnen und -assistenten, Pharmazeutisch-technische Assistentinnen und Assistenten

^b^Diverse aus Analyse ausgeschlossen

^c^„stimme überhaupt nicht/eher nicht/teils teils zu“ als Referenzgruppe

Ein besonders gewichtiger Faktor war das Geschlecht: Die statistische Chance, sich impfen zu lassen, war unter Männern fast doppelt so hoch wie unter Frauen (die Odds Ratios (ORs) sind Tab. 2 zu entnehmen). Die Berufsgruppe der Ärztinnen und Ärzte wies von allen befragten Berufsgruppen im Vergleich mit Personen aus dem Krankenpflegeberuf die höchste Odds Ratio auf.

Wer sich gut informiert fühlte, zeigte eine um 75 % erhöhte statistische Chance, bereit zu einer Impfung zu sein. Wer Angst hatte, sich selbst zu infizieren, hatte eine um 45 % erhöhte Chance, sich impfen lassen zu wollen, wer Angst davor hatte, die Familie anzustecken, eine um 41 % erhöhte Chance. Sich durch die Infektionsschutzmaßnahmen der Behörden geschützt zu fühlen, erhöhte die Impfbereitschaftschance um 64 %, während der wahrgenommene Schutz durch die Maßnahmen des Arbeitgebers sie um 18 % erhöhte. Personen ohne Migrationshintergrund wiesen eine um 30 % höhere Impfbereitschaftschance auf als Personen mit Migrationshintergrund. Sich aufgrund von Alter oder Vorerkrankung einer Risikogruppe zugehörig zu fühlen, erhöhte die statistische Chance auf Impfbereitschaft um 16 %. In der Gruppe, die keine klinisch relevanten Depressionssymptome zeigte, war die Impfbereitschaftschance um 19 % höher als in der Gruppe mit entsprechenden Symptomen.

## Diskussion

Ziel der Studie war ein besseres Verständnis der Bereitschaft zur COVID-19-Impfung unter Beschäftigten im Gesundheitswesen in Deutschland, insbesondere in Abhängigkeit von soziodemografischen, berufsbezogenen und COVID-19-spezifischen Charakteristika sowie der psychischen Gesundheit und psychischen Ressourcen.

Knapp 2 Drittel der Teilnehmenden waren zwischen November 2020 und Januar 2021 zu einer COVID-19-Impfung motiviert. Die Impfbereitschaft lag damit etwas höher als die Ende 2020 berichtete Impfbereitschaft in der Allgemeinbevölkerung, wobei der COVID-19-Monitor Beschäftigten im Gesundheitswesen eine tendenziell eher niedrigere Impfbereitschaft zuschrieb [7]. Möglicherweise spielte die Selbstselektion der Teilnehmenden eine Rolle, insofern dass durch die verschiedenen Rekrutierungswege der Studien Personen mit verschiedenen (Impf‑)Motivationen angesprochen wurden.

Insgesamt stehen die Ergebnisse der Studie in Einklang mit bisherigen Untersuchungen zur COVID-19-Impfbereitschaft. In Bezug auf demografische Merkmale untermauern sie, dass männliches Geschlecht, ein höheres Alter und Kinderlosigkeit mit höherer Impfbereitschaft in Zusammenhang stehen (vgl. z. B. [7, 8, 12, 14, 15]). Eine mögliche Erklärung in Bezug auf das Alter könnte sein, dass mit höherem Alter ein schwerer COVID-19-Verlauf wahrscheinlicher wird [34] und die Impfbereitschaft eher auf die Furcht vor einer Ansteckung zurückzuführen sein könnte. Eventuell spielen bei der niedrigeren Impfbereitschaft von Frauen zumindest in bestimmten (jüngeren) Altersgruppen Überlegungen zu Impfauswirkungen auf (zukünftige) Schwangerschaften eine Rolle.

Dass sich Personen mit Migrationshintergrund geringfügig weniger impfbereit zeigten, kann möglicherweise dadurch erklärt werden, dass ihr Vertrauen in die öffentlichen Abläufe in Deutschland niedriger ist [35] oder sprachliche oder kulturelle Hürden die Informationssuche erschweren [36].

Ärztinnen und Ärzte zeigten die höchste Impfbereitschaft im Vergleich mit den anderen Berufsgruppen. Dies ließe sich dadurch erklären, dass diese Gruppe eine höhere formale Bildung hat [18] und wegen ihres Berufes sehr gut über COVID-19 und die Impfung informiert ist [7, 9, 17].

Im Einklang mit bisherigen Forschungsergebnissen [7, 9, 17] zeigt unsere Studie, dass ein ausreichender Informationsgrad mit einer höheren Impfbereitschaft zusammenhängt, und bestätigt die Bedeutung von gut zugänglichen seriösen Informationen.

Wie erwartet [10] ist auch die Zufriedenheit mit den Infektionsschutzmaßnahmen politischer Instanzen, insbesondere der wahrgenommene Schutz durch diese, deutlich mit einer erhöhten Impfbereitschaft assoziiert. Die Maßnahmen des Arbeitgebers bzw. die Wahrnehmung dieser erhöhen die Impfbereitschaft ebenfalls, wenn auch nicht in einem so deutlichen Ausmaß wie die der nationalen/lokalen Behörden.

Der Befund, dass die Angst, sich selbst oder die Familie zu infizieren, mit einer höheren Impfbereitschaft einhergeht, ist ebenfalls konform mit bisherigen Forschungsergebnissen [15, 17]. Bemerkenswert ist hierbei, dass die spezifische Angst vor einer Ansteckung eine relevante Rolle spielt, nicht aber die generalisierte Angst.

Während es im Hinblick auf die Impfbereitschaft keinen bedeutsamen Unterschied machte, ob klinisch relevante Symptome von generalisierter Angst vorlagen, standen klinisch relevante Depressionssymptome in Zusammenhang mit einer niedrigeren Impfbereitschaft. Das scheint insofern plausibel, da Depressionen mit einer negativen Sicht auf sich selbst, die Zukunft und die Welt einhergehen [37] und somit möglicherweise auch mit einer negativeren Sichtweise auf die Impfung und ihre Vertrauenswürdigkeit.

Die Ergebnisse sind auch vor dem Hintergrund relevant, dass von Mai bis Juli 2020 die psychische Belastung der Allgemeinbevölkerung durch Angst und depressive Symptome anhielt, subjektives Informationslevel, COVID-19-bezogene Angst und Vertrauen in die staatlichen Maßnahmen jedoch abnahmen [20].

### Limitationen

Während eines Großteils des Befragungszeitraumes war in Deutschland noch kein COVID-19-Impfstoff zugelassen, weshalb sich die Frage nach der Impfbereitschaft auf eine hypothetische Zukunft beziehen musste.

Eine andere Einschränkung der vorliegenden Arbeit stellt die querschnittliche Auswertung der prospektiv angelegten VOICE-Studie dar, welche keine Kausalschlüsse zulässt. Sie kann jedoch Zusammenhänge aufzeigen, die aufgrund der großen Stichprobe eine relativ hohe Aussagequalität aufweisen.

Durch die Selbstselektion der Stichprobe im Rahmen der adressierten Beschäftigten des Gesundheitswesens nahmen möglicherweise eher Personen ohne oder mit geringeren sprachlichen und kulturellen Schwierigkeiten und einem höheren Vertrauen in die Wissenschaft teil, sodass die Möglichkeit einer Überschätzung der Impfbereitschaft besteht.

Einschränkend ist zudem, dass die Impfbereitschaft dichotom erfasst wurde und einige Variablen, wie z. B. die der psychischen Gesundheit, mit wenigen Items erhoben wurden. Dies reduzierte möglicherweise die Kriteriumsvalidität, erhöhte jedoch die Ökonomie und Benutzerfreundlichkeit der Befragung dieser beruflich durch die Pandemie im besonderen Maße geforderten Zielgruppe.

### Implikationen

Die vorliegende Studie konnte bestehendes Wissen über Faktoren, die mit einer COVID-19-Impfbereitschaft in Verbindung stehen, anhand einer großen Stichprobe von 6217 Beschäftigten im Gesundheitswesen in Deutschland bestätigen und dieses erweitern, indem neben der spezifischen Angst vor einer SARS-CoV-2-Infektion auch generalisierte Angstsymptome sowie Depressionssymptome in den Kontext eingebracht wurden.

Die Ergebnisse der Studie unterstreichen die Bedeutung von Informationen über die COVID-19-Erkrankung sowie über die Schutzimpfung, auch für im Gesundheitswesen Tätige. Politik und Arbeitgeber können nicht davon ausgehen, dass Personen mit Gesundheitsberufen automatisch über ausreichende Informationen verfügen (vgl. [7]). Politik und Arbeitgeber sollten stattdessen aktiv informieren und darauf achten, dass die Informationen alle Gruppen erreichen und dabei auch potenzielle sprachliche und kulturelle Hürden überwinden.

Die Ergebnisse untermauern außerdem, dass der wahrgenommene Schutz durch Infektionsschutzmaßnahmen politischer Instanzen oder der Arbeitgeber für Beschäftigte im Gesundheitswesen deutlich zur Impfbereitschaft beizutragen scheint. Ein erkennbar verantwortungsvoller Umgang mit der gesundheitlichen Sicherheit der im Gesundheitsbereich Tätigen könnte demnach auch die Impfbereitschaft erhöhen.

Da Anzeichen einer Depression mit einer niedrigeren Impfbereitschaft zusammenhängen, könnte die Prävention von Depressionen, beispielsweise mithilfe von psychosozialen Unterstützungsangeboten, für die Impfbereitschaft vorteilhaft sein.

In Anbetracht der möglichen Notwendigkeit einer Auffrischungsimpfung [38] gewinnen die Ergebnisse für den weiteren Verlauf der COVID-19-Pandemie in Deutschland zusätzlich an Bedeutung.

## Fazit

- Männer, Personen über 40 Jahre und diejenigen, die Angst davor haben, sich selbst oder die Familie zu infizieren, scheinen auch unter Beschäftigten des Gesundheitswesens in Deutschland eine höhere COVID-19-Impfbereitschaft aufzuweisen als Vergleichsgruppen.
- Ärztinnen und Ärzte sind die Berufsgruppe mit der höchsten Impfbereitschaft.
- Ausreichende Informationen über COVID-19 hängen mit einer höheren Impfbereitschaft zusammen. Seriöse, niederschwellige und eventuell mehrsprachige Informationen über die Krankheit und die Impfung sollten unterstützt werden.
- Wer sich durch die Infektionsschutzmaßnahmen politischer Instanzen und der Arbeitgeber geschützt fühlt, scheint eher einer Impfung zuzustimmen, was die Relevanz eines verantwortungsvollen Umgangs mit der Sicherheit der im Gesundheitsbereich Tätigen unterstreicht.
- Prävention von Depressionssymptomen beim Gesundheitspersonal könnte auch im Kontext von Impfbereitschaft vorteilhaft sein.
